# Assessment of a novel semi-automated dataset for large-scale surveillance of central venous catheter–related mechanical complications

**DOI:** 10.1093/intqhc/mzag080

**Published:** 2026-06-05

**Authors:** Emilia Ängeby, Maria Adrian, Mathias Lazarevic Lindblad, Ola Borgquist, Thomas Kander

**Affiliations:** Department of Clinical Sciences, Lund University, Lund, Sweden; Department of Intensive and Perioperative Care, Skåne University Hospital, Lund, Sweden; Department of Clinical Sciences, Lund University, Lund, Sweden; Department of Anaesthesia and Perfusion, The Prince Charles Hospital, Brisbane, Queensland, Australia; Department of Clinical Sciences, Lund University, Lund, Sweden; Department of Intensive and Perioperative Care, Skåne University Hospital, Lund, Sweden; Department of Clinical Sciences, Lund University, Lund, Sweden; Department of Cardiothoracic Surgery, Anaesthesia and Intensive Care, Skåne University Hospital, Lund, Sweden; Department of Clinical Sciences, Lund University, Lund, Sweden; Department of Intensive and Perioperative Care, Skåne University Hospital, Lund, Sweden

## Abstract

**Background:**

The aim of this study was to examine whether a novel semi-automated dataset based on electronic health record documentation can be used for surveillance of central venous catheter-related mechanical complications (failed catheterization, bleeding, cardiac arrhythmia, pneumothorax and nerve injury) within 24 h of catheterization.

**Methods:**

The semi-automated dataset comprised a fully automated extraction of clinical documentation from the electronic health record supplemented with a minor manual review aimed at identifying pneumothoraces, as these are rarely diagnosed at the time of insertion but rather after a postprocedural chest X-ray. To assess surveillance performance, we compared the number of mechanical complications between the semi-automated and manually evaluated datasets for the same cohort and study period, focusing on agreement in aggregate counts. Comparisons were made at the group level only, without enforcing insertion-by-insertion matching.

**Results:**

A total of 12 667 insertions were included. Minor mechanical complications occurred in 615 (4.9%) of the insertions in the semi-automated dataset and in 645 (5.1%) of the insertions in the manually validated dataset. Major mechanical complications occurred in 44 (0.35%) of the insertions in the semi-automated dataset compared to 48 (0.38%) in the manually validated dataset.

**Conclusion:**

A semi-automated dataset based on electronic health record documentation provides sufficiently accurate surveillance of central catheterization-related mechanical complications at the group level. Despite minor discrepancies, the semi-automated method enhances efficiency, scalability, and supports continuous real-time quality assurance. The potential underestimation of complication rates is offset by the possibility of robust real-time quality assurance in registries and a substantial analytical power in scientific studies.

## Introduction

Central venous catheters (CVCs) play an essential role in modern healthcare. Unfortunately, they are associated with complications: mechanical, infectious, and thrombotic [1, 2]. In the present study, we focus on mechanical complications. These often arise within 24 h after the insertion and include bleeding, arterial puncture/catheterization, nerve injury, cardiac arrhythmias, pneumothorax, and tip malposition [3–6]. Previous studies have shown that CVC-related complications occur in 2%–10% of all insertions and result in increased costs, longer patient in-hospital stays, increased mortality and increased patient suffering [7–9]. Thus, it is of utmost importance to minimize the risk of complications.

Documentation and follow-up actions of insertions and CVC management are important in order to reduce and prevent complications [10]. Accordingly, both the Association of Anaesthetists of Great Britain and Ireland and the American Society of Anaesthesiologists Task Force on Central Venous Access have published protocols for documentation of insertions and complications management [11, 12]. The Swedish Association of Local Authorities and Regions and the Swedish Society of Anaesthesia and Intensive Care recommend that all departments where CVCs are inserted should perform continuous quality controls [13].

Manually extracted data are often considered the gold standard in retrospective, observational studies [14, 15]. In a previous study from our group (CVC-MECH), we conducted a manual review of an automated extraction of data on 12 667 CVC insertions collected from *InformationsPlattformen Region Skåne* regarding CVC-related mechanical complications (i.e. failed catheterization, bleeding, arrhythmia, pneumothorax, and nerve injury) [16]. *InformationsPlattformen* is a comprehensive database that integrates real-time data from the electronic health records (EHR) within a large digital infrastructure. Through this platform, we have access to data on all CVC insertions in Region Skåne, Sweden (four acute care hospitals and one university hospital).

Despite the importance of monitoring mechanical complications associated with CVC insertions, large-scale surveillance remains challenging. Manual chart review and case validation are resource-intensive and limit the feasibility of timely, high-powered studies. A semi-automated approach using routinely collected electronic health record data could offer the potential to improve efficiency and scalability. However, the validity for identifying CVC-related mechanical complications using a more automated approach has not been sufficiently evaluated. Addressing this gap is essential to determine whether a semi-automated approach can reliably support large-scale quality monitoring and research regarding CVC-related mechanical complications.

The aim of this study was to examine whether a new semi-automated dataset supplemented by a minor manual review can be used for surveillance of CVC-related mechanical complications. The primary performance criterion was agreement in aggregate counts compared with a manually evaluated reference dataset, CVC-MECH trial [16]. We compared the number of CVC-related mechanical complications between the semi-automated and ­manually evaluated datasets for the same cohort and time window, using aggregate cohort-level counts rather than matching individual patients or catheters between datasets.

## Materials and methods

The study was approved by the Swedish Ethical Review Authority (Dnr 2018/295). No interventions were included in the study, and the Authority waived the requirement for informed consent. The CVC-MECH trial was registered at ClinicalTrials.gov (NTC3782324), and the manuscript was written according to the STROBE guidelines for observational studies (Supplementary Appendix, pp. 1–3). Patients were not involved in the design, conduct, reporting, or dissemination of this study. However, the study was developed with the aim of improving patient safety and care quality by enabling more efficient identification of CVC-related complications. The findings are intended to support clinical decision-making and enhance quality assurance processes in the interest of current and future patients.

We included all CVC insertions in patients ≥16 years performed in Region Skåne, Sweden (serving 1.4 million people) from 2 March 2019 until 31 December 2020. The number of minor and major mechanical complications in the semi-automated dataset was compared with the number of minor and major mechanical complications in the manually validated CVC-MECH dataset, which contained CVC insertions from the same Region and study period [16].

### Data collection: semi-automated dataset

The semi-automated dataset was based on clinical documentation in the EHR exported to *InformationsPlattformen* followed by automatic extraction along with a minor manual review.

*InformationsPlattformen* is a digital information infrastructure made to manage and consolidate patient data records from all hospitals within Region Skåne. It enables clinicians and other healthcare providers to gain access to continuously updated patient data and information. *InformationsPlattformen* integrates information through connections with different healthcare systems, including the EHR system (Melior^TM^, Cerner Corporation, North Kansas City, Missouri, USA), the radiology information system (Sectra, Sectra Medical, Linköping, Sweden), and the laboratory information systems (LIMS Software Point, Tel Aviv, Israel). Data export was performed using secure interfaces and standardized data exchange protocols.

The fully automated extraction included insertion-related data encompassing patient- and catheter baseline variables, as well as immediate mechanical complications, all of which were documented in the same CVC insertion template in the EHR (Supplementary Appendix, pp. 4–5).

The minor manual review was performed to increase the chances of identifying CVC-related pneumothoraces. These were not always found in the insertion template (i.e. in the automated extraction) as the diagnosis generally was established only after a postprocedural chest X-ray. A separate data extraction was performed through *InformationsPlattformen,* including all radiology reports of chest investigations conducted from 4 h prior to 24 h after CVC insertion that contained the term 'pneumothorax' (including common spellings and misspellings of the word) using a rule-based keyword search approach. Radiology reports that negated pneumothorax were excluded using a predefined rule, provided a negation was found within 40 characters prior to the term 'pneumothorax’. The pre-insertion interval was included to account for potential imprecision in documented insertion times. The post-insertion interval was chosen since the definition of mechanical complications is within 24 h after insertion. Two independent reviewers (EÄ and TK) assessed the dataset and identified CVC-related pneumothoraces based on the full radiology report. A manual review of the EHR was conducted if any doubts arose.

### Outcomes

The outcomes were the number of mechanical complications within 24 hours after central venous catheterization, which included: failed catheterization, bleeding and/or arterial catheterization or puncture, cardiac arrhythmias, pneumothorax, and nerve injury. Cardiac arrhythmias and bleeding were further graded according to the Common Terminology Criteria for Adverse Events (version 6.0) [17]. Bleeding grade 1 refers to self-limiting oozing bleeding, which is rarely documented and is not included in the current study. Bleeding grade 2 refers to bleeding in need of external compression. Given that arterial puncture with the steel canula often results in grade 2 bleeding, arterial puncture was also classified as a grade 2 bleeding in this study to ensure consistency and accurately reflect its clinical significance. Bleeding grade 3 refers to bleeding that necessitates a red blood cell transfusion or surgical intervention, whereas a grade 4 bleeding is considered life-threatening. Arterial catheterization was classified as a grade 3 bleeding in this study. Arrhythmias grade 1 and 2 refer to asymptomatic or self-limiting arrhythmias that do not require urgent medical intervention, whereas grades 3 and 4 refer to symptomatic or life-threatening arrhythmias in need of urgent medical treatment. The classification of mechanical complications used in this study is presented in Table 1.

**Table 1:** Classification of minor and major mechanical complications used in the current study.

Minor mechanical complications	Major mechanical complications
Failed catheterization	Bleeding grades 3–4^a^
Bleeding grade 2^b^	Arrhythmia grades 3–4^c^
Arrhythmia grades 1–2^d^	Pneumothorax
Non-persistent nerve injury^e^	Persistent nerve injury^f^

aArterial catheterization or bleeding in need of blood transfusion, invasive intervention, or life-threatening bleeding.

bArterial puncture or bleeding in need of external compression.

cSymptomatic or life-threatening arrhythmia in need of urgent medical intervention.

dNon-symptomatic, self-limiting, or arrhythmia not needing urgent medical intervention.

eSymptomatic nerve injury lasting <72 h.

fSymptomatic nerve injury lasting ≥72 h.

Bleeding grade 2, arrhythmia grades 1–2, failed catheterization, and non-persistent nerve injury were classified as minor mechanical complications. Bleeding grades 3–4, arrhythmia grade 3–4, pneumothorax, and persistent nerve injury were classified as major mechanical complications.

The number of outcomes in the semi-automated dataset was compared to the number of outcomes in the manually validated dataset from the CVC-MECH trial. Baseline characteristics were the same for both datasets.

### Statistics

Sample size was established in the main trial [16]. Normally distributed continuous variables are reported as mean and standard deviation. Non-normally distributed variables are presented as median and interquartile range (IQR). Binary and categorical variables are presented as numbers and percentages. The number of minor and major mechanical complications is reported as counts and percentages. One CVC insertion can be associated with more than one complication.

Absolute differences were calculated using *MedCalc* from MedCalc Software (MedCalc Software Ltd, Ostend, Belgium). Excel (version 16.79.2, Microsoft Corporation, Redmond, Washington, USA) was used to compare the two datasets. A *P-*value <.05 was considered statistically significant.

## Results

The main trial shows a flowchart illustrating the selection process for CVC insertions [16].

CVC baseline and insertion characteristics are presented in Table 2. A total of 12 667 insertions in 8586 patients were included. Among these, 39% of the insertions were in female patients. Most (81%) insertions were performed at a university hospital. More than half of the insertions (53%) took place in an operating theatre, 60% were carried out by male operators, and 86% of the CVCs were ≥15 cm in length.

**Table 2:** Baseline characteristics per CVC.

CVC insertions, *n* (%)	12 667 (100)
Number of patients, *n*	**8586**
Female sex, *n* (%)	4903 (39)
Age (years), mean (SD)	65 (15)
BMI, mean (SD)	27 (5.9)
Missing	323 (2.5)
Coagulopathy at insertion,^a^ *n* (%)	1927 (15)
Invasive positive-pressure ventilation, *n* (%)	6449 (51)
Admitted to ICU, *n* (%)	5538 (44)
Hospital for insertion, *n* (%)	
University hospital	10 223 (81)
Non-university hospital	2182 (17)
Missing	262 (2.1)
Room for insertion, *n* (%)	
Operating theatre	6650 (53)
ICU and other wards	5444 (43)
Missing	573 (4.5)
Nightly insertions,^b^ *n* (%)	1640 (13)
Operator gender, *n* (%)	
Female operator	4511 (36)
Male operator	7640 (60)
Missing	516 (4.1)
Insertion technique, *n* (%)	
Ultrasound, in-plane	2611 (21)
Ultrasound, in-plane/out-of-plane	377 (3.0)
Ultrasound, out-of-plane	8437 (67)
Ultrasound, before insertion only	383 (3.0)
Landmark	318 (2.5)
Change over guidewire	167 (1.3)
Missing	374 (3.0)
Insertion site, *n* (%)	
Internal jugular vein	10 464 (83)
External jugular vein	85 (0.7)
Subclavian vein	1602 (13)
Femoral vein	237 (1.9)
Missing	279 (2.2)
Number of skin punctures, median (IQR)	1 (1)
Number of lumens, *n* (%)	
1	3280 (26)
2	2953 (23)
3	3783 (30)
4	865 (7.1)
5	1321 (10)
Missing	465 (3.7)
Catheter length, *n* (%)	
<15 cm	1085 (8.6)
≥15 cm	10 830 (86)
Missing	752 (5.9)

aPlatelet count <50 × 10^9^ l^−1^, prothrombin time/international normalized ratio >1.8 or activated partial thromboplastin time >1.3 × normal value (43 s).

bInsertions performed between 21:00 and 07:00.

All outcomes for both the semi-automated dataset and the manually validated dataset in CVC-MECH [16] are reported in Table 3. There were no substantial differences in the numbers of different outcomes between the datasets. Minor mechanical complications occurred in 615 (4.9%) of the insertions in the semi-automated dataset and in 645 (5.1%) of the insertions in the CVC-MECH dataset, absolute difference 0.24% (95% CI −0.30 to 0.77). Major mechanical complications occurred in 44 (0.35%) of the insertions in the data from the semi-automated dataset, compared to 48 (0.38%) of the insertions in CVC-MECH, with an absolute difference 0.032% (95% CI −0.12 to 0.18). Bleeding grade 3–4 was the most common major complication in both datasets (0.14% in the semi-automated dataset and 0.17% in CVC-MECH), followed by pneumothorax (0.14% in the semi-automated dataset and 0.13% in CVC-MECH). A total of 2283 radiology reports were reviewed in the separate pneumothorax extraction. Of these, 45 radiology reports and corresponding EHRs were manually reviewed, identifying 13 additional CVC-related pneumothoraces beyond the five recorded in the EHR template, for a total of 18 pneumothoraces in the semi-automated dataset. In the CVC-MECH dataset, 17 pneumothoraces were identified.

**Table 3:** Total number of outcomes per dataset.

	**Semi-automated dataset ** **n = 12 667 n, (%)**	CVC-MECH n = 12 667 n, (%)	Absolute difference (95% confidence interval)	P-value
**Minor complications**	**615 (4.9)**	**645 (5.1)**	**0.24 (−0.30 to 0.77)**	**.39**
** Failed catheterization**	89 (0.70)	91 (0.72)	0.016 (**−**0.19 to 0.22)	.88
** Bleeding grade 2^a^**	461 (3.6)	490 (3.9)	0.23 (**−**0.24 to 0.70)	.34
** Cardiac arrhythmia grades 1–2^b^**	65 (0.51)	64 (0.51)	0.0080 (**−**0.18 to 0.18	.93
** Non-persistent nerve injury^c^**	–	–	–	–
**Major complications**	**44 (0.35)**	**48 (0.38)**	**0.032 (−0.12 to 0.18)**	**.68**
** Bleeding grades 3–4^d^**	18 (0.14)	21 (0.17)	0.024 (**−**0.073 to 0.012)	.63
** Cardiac arrhythmia grades 3–4^e^**	8 (0.063)	9 (0.071)	0.0080 (**−**0.056 to 0.072)	.81
** Pneumothorax**	18 (0.14)	17 (0.13)	0.0080 (**−**0.014 to 0.030)	.87
** Persistent nerve injury^f^**	–	1 (0.0079)	0.0080 (**−**0.014 to 0.030)	.50

aArterial puncture or bleeding in need of external compression.

bNon-symptomatic, self-limiting, or arrhythmia not needing urgent medical intervention.

cSymptomatic nerve injury lasting <72 h.

dArterial catheterization or bleeding in need of blood transfusion, invasive intervention, or life-threatening bleeding.

eSymptomatic or life-threatening arrhythmia in need of urgent medical intervention.

fSymptomatic nerve injury lasting ≥72 h.

## Discussion

### Statement of principal findings

This study demonstrates that semi-automated data extraction can provide reliable group-level surveillance of CVC-related mechanical complications, with only small, statistically non-significant differences between datasets, indicating strong agreement in aggregate complication counts across surveillance periods. To the best of our knowledge, this is the first study to compare a semi-automated dataset to a manually reviewed dataset regarding CVC-related mechanical complications. Semi-automated extraction enhances scalability, efficiency, and analytical power for large datasets, enabling real-time tracking of performance indicators and identification of agreement in aggregated complication counts. The small discrepancies observed between datasets are acceptable in a surveillance context, where agreement in aggregate counts is more important than perfect case-by-case alignment.

The manually validated dataset used as the reference in this study, the CVC-MECH study, included 12 667 CVC insertions and is currently the largest study on CVC-related complications [16]. As of November 2025, *InformationsPlattformen* contains data on approximately 45 400 CVC insertions. With more than 7000 insertions added annually, this will be the largest existing database on CVC insertions and enable both comprehensive research and a high level of quality assurance as required by regulatory authorities. While semi-automated extraction may result in a minor loss of precision, this is offset by a substantial increase in statistical power and the ability to support continuous clinical quality control through large-scale, real-time surveillance of complication counts.

The semi-automated dataset identified 30 fewer minor and four fewer major mechanical complications than the manually validated dataset, corresponding to absolute differences of 0.24 (95% CI −0.30 to 0.77) and 0.032 (95% CI −0.12 to 0.18) percentage points, respectively. A semi-automated extraction may thus somewhat underestimate complication rates. Although it is important to prevent and reduce the occurrence of minor complications, these are rarely significant for the patients, whereas major complications are associated with significant morbidity and mortality [8].

### Strengths and limitations

This study demonstrates that a semi-automated dataset based on EHR documentation can be effectively used for surveillance of central venous catheter–related mechanical complications. The agreement in aggregate counts is sufficient to support routine surveillance, large-scale research, and continuous quality improvement. Our findings provide the possibility of robust real-time quality assurance in registries and substantial analytical power in scientific studies.

Nonetheless, this study has several limitations. First, there is a risk that not all CVC insertions were documented and, in some cases, not reported in a correct manner. Second, the primary comparison relied on manually evaluated data, which might not be entirely accurate. Human errors, inconsistencies in manual data review, and inter-rater variability could have introduced biases and inaccuracies, potentially affecting the reliability of the manually evaluated dataset. Third, some mechanical complications may have been identified later than within the first 24 h post-insertion, and therefore not reported in the insertion template in the EHR.

### Interpretation within the context of the wider literature

Previous studies have demonstrated that electronically extracted data can achieve a quality comparable to manually collected data [15, 18]. Brundin-Mather *et al.* [18] reported a high degree of concordance between data derived from an integrated critical care information system and manually recorded bedside data, with minor discrepancies largely attributable to inconsistencies in manual documentation rather than limitations of electronic extraction. Similarly, Arts *et al.* [15] highlighted that structured and standardized data collection improves registry accuracy and reduces variability, supporting the use of electronic methods for clinical quality monitoring.

Our findings are consistent with these observations. The semi-automated dataset in the present study showed strong agreement in aggregate complication counts compared to the manually evaluated dataset, suggesting that electronic extraction methods can reliably capture clinically meaningful outcomes even in complex procedural settings such as central venous catheterization. As demonstrated by Arts *et al.* [15] in the Dutch NICE registry, electronically collected data can, in some cases, outperform manually abstracted data when validated against gold-standard re-abstraction. While manual review is prone to random errors related to transcription or interpretation, semi-automated methods mainly introduce systematic, programming-related errors that are easier to detect and correct.

### Implications for policy, practice, and research

Taken together, these results reinforce that semi-automated extraction offers a robust and scalable approach for both research and continuous clinical quality control. By maintaining high data integrity at the group level and enabling efficient real-time surveillance, such systems can substantially strengthen patient safety monitoring and quality assurance in healthcare.

It has previously been suggested that electronic data processing methods may improve research and quality assurance while reducing costs and data entry errors [19–22]. Pavlovic *et al.* [22] also suggested that electronic methods will lead to reduced costs and lower source-to-database error rates. According to the national guidelines for the management and insertion of CVCs, as set by the Swedish National Board of Health and Welfare, all CVC insertions must be monitored regarding potential complications. Our semi-automated data extraction method could facilitate this, reallocating resources to patient care rather than manual data review, ultimately lowering costs and enhancing healthcare ­quality [13].

Although the present study is based on a rule-based semi-automated extraction approach, future developments may involve artificial intelligence (AI) and large language models to further enhance medical documentation and research. Dahl Haue *et al.* [23] highlighted AI’s role in large-scale data analysis and early cancer detection. In CVC research, AI-driven natural language processing can extract relevant information from unstructured EHRs, reducing the need for manual chart reviews. Such approaches may allow the semi-automated dataset to be further developed into a fully automated extraction in future work, with the potential to improve data quality and utilization.

### Conclusions

In this study, we have demonstrated that a semi-automated dataset, based on EHR documentation, can be effectively used for surveillance of CVC-related mechanical complications. While minor differences compared to manually evaluated data exist, agreement in aggregate counts is sufficient to support routine surveillance, large-scale research, and continuous quality improvement. Further AI integration could improve precision, supporting EHR-extraction.

## Supplementary Material

mzag080_Supplementary_Data

## Data Availability

The data that support the findings of this study originate from the Region Skåne electronic health record system and are subject to regional data protection regulations. Aggregated, de-identified data may be made available from the corresponding author upon reasonable request and with permission from the data owner.

## References

[1] Polderman KH , Girbes AJ. Central venous catheter use. Part 1: mechanical complications. Intensive Care Med 2002;28:1–17. 11818994 10.1007/s00134-001-1154-9

[2] Polderman KH , Girbes AR. Central venous catheter use. Part 2: infectious complications. Intensive Care Med 2002;28:18–28. 11818995 10.1007/s00134-001-1156-7

[3] McGee DC , Gould MK. Preventing complications of central venous catheterization. N Engl J Med 2003;348:1123–33. 12646670 10.1056/NEJMra011883

[4] Pikwer A , Baath L, Davidson B et al The incidence and risk of central venous catheter malpositioning: a prospective cohort study in 1619 patients. Anaesth Intensive Care 2008;36:30–7. 18326129 10.1177/0310057X0803600106

[5] Eisen LA , Narasimhan M, Berger JS et al Mechanical complications of central venous catheters. J Intensive Care Med 2006;21:40–6. 16698743 10.1177/0885066605280884

[6] Angeby E , Adrian M, Bozovic G et al Central venous catheter tip misplacement: a multicentre cohort study of 8556 thoracocervical central venous catheterisations. Acta Anaesthesiol Scand 2024;68:520–9. 38351546 10.1111/aas.14380

[7] Bjorkander M , Bentzer P, Schott U et al Mechanical complications of central venous catheter insertions: a retrospective multicenter study of incidence and risks. Acta Anaesthesiol Scand 2019;63:61–8. 29992634 10.1111/aas.13214

[8] Ingefors S , Adrian M, Heckley G et al Major immediate insertion-related complications after central venous catheterisation and associations with mortality, length of hospital stay, and costs: a prospective observational study. J Vasc Access 2025;26:487–96. 38267828 10.1177/11297298231222929 PMC11894852

[9] Rahmqvist M , Samuelsson A, Bastami S et al Direct health care costs and length of hospital stay related to health care-acquired infections in adult patients based on point prevalence measurements. Am J Infect Control 2016;44:500–6. 26988332 10.1016/j.ajic.2016.01.035

[10] Campanella P , Lovato E, Marone C et al The impact of electronic health records on healthcare quality: a systematic review and meta-analysis. Eur J Public Health 2016;26:60–4. 26136462 10.1093/eurpub/ckv122

[11] Bodenham Chair A , Babu S, Bennett J et al Association of anaesthetists of Great Britain and Ireland: safe vascular access 2016. Anaesthesia 2016;71:573–85. 26888253 10.1111/anae.13360 PMC5067617

[12] Practice guidelines for central venous access 2020: an updated report by the American Society of Anesthesiologists Task Force on Central Venous Access. Anesthesiology 2020;132:8–43. 31821240 10.1097/ALN.0000000000002864

[13] Frykholm P , Pikwer A, Hammarskjold F et al Clinical guidelines on central venous catheterisation. Swedish Society of Anaesthesiology and Intensive Care Medicine. Acta Anaesthesiol Scand 2014;58:508–24. 24593804 10.1111/aas.12295

[14] Yin AL , Guo WL, Sholle ET et al; Weill Cornell COVID-19 Data Abstraction Consortium. Comparing automated vs. manual data collection for COVID-specific medications from electronic health records. Int J Med Inform 2022;157:104622. 34741892 10.1016/j.ijmedinf.2021.104622 PMC8529289

[15] Arts DG , De Keizer NF, Scheffer GJ. Defining and improving data quality in medical registries: a literature review, case study, and generic framework. J Am Med Inform Assoc 2002;9:600–11. 12386111 10.1197/jamia.M1087 PMC349377

[16] Adrian M , Borgquist O, Kroger T et al Mechanical ­complications after central venous catheterisation in the ultrasound-guided era: a prospective multicentre cohort study. Br J Anaesth 2022;129:843–50. 36280461 10.1016/j.bja.2022.08.036

[17] National Cancer Institute CTEP. Common terminology criteria for adverse events (CTCAE) version 6.0 (MedDRA v28.0). Bethesda, MD: U.S. Department of Health and Human Services. https://dctd.cancer.gov/research/ctep-trials/for-sites/adverse-events (October 2025, last accessed).

[18] Brundin-Mather R , Soo A, Zuege DJ et al Secondary EMR data for quality improvement and research: a comparison of manual and electronic data collection from an integrated critical care electronic medical record system. J Crit Care 2018;47:295–301. 30099330 10.1016/j.jcrc.2018.07.021

[19] Edwards JR , Pollock DA, Kupronis BA et al Making use of electronic data: the national healthcare safety network eSurveillance initiative. Am J Infect Control 2008;36:S21–6. 18374208 10.1016/j.ajic.2007.07.007

[20] Eisenstein EL , Collins R, Cracknell BS et al Sensible approaches for reducing clinical trial costs. Clin Trials 2008;5:75–84. 18283084 10.1177/1740774507087551

[21] Yip A , Leduc M, Teo V et al A novel method to link and validate routinely collected emergency department clinical data to measure quality of care. Am J Med Qual 2009;24:185–91. 19372541 10.1177/1062860609333880

[22] Pavlovic I , Kern T, Miklavcic D. Comparison of paper-based and electronic data collection process in clinical trials: costs simulation study. Contemp Clin Trials 2009;30:300–16. 19345286 10.1016/j.cct.2009.03.008

[23] Haue AD , Hjaltelin JX, Holm PC et al Artificial intelligence-aided data mining of medical records for cancer detection and screening. Lancet Oncol 2024;25:e694–703. 10.1016/S1470-2045(24)00277-8 39637906

